# Acupuncture combined with moxibustion for insomnia after stroke: A protocol for systematic review and meta analysis

**DOI:** 10.1097/MD.0000000000024112

**Published:** 2021-01-22

**Authors:** Junjun Sun, Zheng Zuo, Ran Song, Xiongying Bao, Miansheng Zhu

**Affiliations:** a Beijing University of Chinese Medicine, Beijing; b Yunnan University of Chinese Medicine; c Kunming St. John's Rehabilitation Hospital; d Second Affiliated Hospital of Yunnan University of Chinese Medicine, Kunming, China; e Institute of Time-Acupoints-Space Acupuncture, Paris, France.

**Keywords:** acupuncture, insomnia after stroke, moxibustion, protocol, system review

## Abstract

**Background:**

Stroke is the main cause of death and disability in the world and insomnia is a common complication of stroke patients. Insomnia will not only seriously affect the prognosis and quality of life of patients with stroke, but even cause the recurrence of stroke. Many studies have proved that acupuncture and moxibustion can effectively improve insomnia symptoms. This study will systematically evaluate the effectiveness and safety of acupuncture combined with moxibustion in treating insomnia after stroke.

**Methods:**

The following 8 databases will be searched from the inception to October 31, 2020, including China National Knowledge Infrastructure Database (CNKI), Chinese Scientific Journal Database (VIP database), China Biomedical Literature Database (CBM), Wanfang Data Chinese Database, PubMed, the Cochrane Central Register of Controlled Trials (CENTRAL), Allied and Alternative Medicine Database (AMED), Excerpt Medica Database (Embase). We will also search for ongoing trials from the World Health Organization International Clinical Trial Registration Platform search portal, Chinese Clinical Trial Register, Clinical trials.gov. In addition, the reference lists of studies meeting the inclusion criteria will also be searched for achieving the comprehensive retrieval to the maximum. All randomized controlled trials of acupuncture and moxibustion in treating insomnia after stroke will be included. Two reviewers will conduct literature screening, data extraction, and quality evaluation respectively. The main outcome is the Pittsburgh sleep quality index (PSQI), and the secondary outcomes include clinical efficacy, quality of life, and safety. RevMan V.5.4.1 will be used for meta-analysis. We will express the results as risk ratio (RR) with 95% confidence intervals (CIs) for dichotomous data and mean difference (MD) or standard mean difference (SMD) 95% CIs for continuous data.

**Results:**

This study will provide a comprehensive review of the available evidence of acupuncture combined with moxibustion in treating insomnia after stroke.

**Conclusion:**

The conclusion of our study will provide the updated evidence to judge the effectiveness and safety of acupuncture combined with moxibustion for the treatment of insomnia after stroke.

**Trial registration number:**

PROSPERO CRD42020216720.

## 1 Introduction

### 1.1 Description of the condition

Stroke, also known as stroke, is an acute cerebrovascular disease, mainly including ischemic stroke and hemorrhagic stroke, which is mainly manifested by the symptoms of focal neurological deficits such as sudden weakness on one side of the face, arms or legs, sudden fainting, and unconsciousness.^[1,2]^ Ischemic stroke is the most common type of stroke, accounting for about 87% of all strokes.^[3,4]^ Stroke has high rates of morbidity, mortality, disability, and recurrence. According to global stroke data, Italy had the highest incidence of stroke from 2004 to 2008, reaching 212/100,000 population per year.^[5,6]^ In the world, stroke is the second leading cause of death after cardiovascular disease.^[7,8]^ In developing countries, stroke is the second leading cause of disability after ischemic heart disease, and the third major cause of disability, after ischemic heart disease and lower back and neck pain in developed countries.^[9]^ The recurrence rate of stroke is approximately 25%.^[10]^ Stroke can cause many complications, mainly including internal medicine diseases and nervous system diseases.^[11]^ Insomnia is one of the common complications of stroke, and the incidence of insomnia after stroke is as high as 38.2%, which is mainly manifested as difficulty in falling asleep and maintaining sleep, waking up early, and being unable to sleep again.^[12,13]^ For patients with stroke, insomnia not only aggravates the physical symptoms, seriously affects the prognosis and quality of life, but may also lead to negative emotions such as anxiety and depression, trigger mental disorders, accelerate the decline of cognitive function, and even cause the recurrence of stroke, thus increasing the economic burden of families and society.^[14,15]^ Medication and cognitive behavioral therapy are mainly adopted for patients with insomnia after stroke at present. However, studies have shown that long-term use of benzodiazepines and other similar drugs will lead to drug dependence, drowsiness, vertigo, cognitive impairment, and other side effects. Patients’ compliance with cognitive behavioral therapy is low. Patients are urgent to seek other effective alternative therapies.^[16–18]^

### 1.2 Description of the intervention

Acupuncture and moxibustion, important components of traditional Chinese medicine (TCM) therapies, have a history of thousands of years in China. They were first spread to Asian countries such as Japan, and then propagated to European and American countries. Due to the characteristics of superior efficiency and low cost, acupuncture and moxibustion have gradually attracted widespread attention from clinicians and researchers in China and abroad.^[19]^ Acupuncture is an external therapy that exerts the therapeutic effect mainly by inserting needles into specific acupoints or regions of the human body. Moxibustion is a kind of external treatment method using moxa as the main raw material, which is ignited and then placed on acupoints or diseased sites for fumigation and ironing, in order to prevent and treat diseases through warm stimulation and drug action. With the development of clinical application, in addition to pure acupuncture, acupuncture therapy also includes electroacupuncture, auricular acupuncture, fire acupuncture, acupoint injection, acupoint catgut embedding, and so on. Moxibustion mainly includes moxa stick moxibustion, moxa cone moxibustion, and moxibustion with moxibustioner (a device in which lighted moxa or moxa stick is placed). Acupuncture and moxibustion have a wide range of indications, which are suitable for internal, external, gynecological, and pediatric departments. The World Health Organization proposed 40 effective diseases for acupuncture and moxibustion in 1980, which increased to 107 in 2002.^[20]^ In 2006, Du et al^[21]^ carried out a study on modern disease menu of acupuncture and moxibustion therapy in China, and the results showed that acupuncture and moxibustion therapy can play a therapeutic role on 461 diseases. In order to improve the clinical efficacy, doctors gradually began to try to apply the combination of acupuncture and moxibustion to treat diseases. The system review conducted by Wang et al^[22]^ showed that the effect of acupuncture combined with moxibustion in the treatment of chronic fatigue syndrome was significantly superior to that of acupuncture or moxibustion alone. Many clinical studies have shown that acupuncture combined with moxibustion is effective in treating insomnia after stroke.^[23–25]^

### 1.3 How the intervention might work

In TCM theory, mechanism of acupuncture may be to regulate meridian qi and dredge meridians, harmonize yin, and yang to transform the body from the imbalance of yin and yang into a balanced state, and reinforce Zheng-qi (healthy energy) and dispel Xie-qi (pathogenic factors). In western medicine, the mechanism of acupuncture is still not very clear.^[19]^ The results of some studies have suggested that the efficacy of acupuncture may be related to the regulation of nervous, endocrine, and immune systems.^[26]^ The startup of moxibustion warming and dredging function is based on rising temperature of the local acupoint.^[27]^ At present, there is a relative lack of research on the mechanism of acupuncture combined with moxibustion for the treatment of insomnia after stroke.

### 1.4 Why it is important to do this review

Acupuncture and moxibustion are both TCM therapies, which are widely used in the treatment of stroke and insomnia. The effectiveness of acupuncture and moxibustion on stroke and insomnia has been confirmed by system reviews.^[28–31]^ Insomnia is a common complication of patients with stroke. Many studies have shown that acupuncture therapy is effective in treating insomnia after stroke.^[32]^ In order to improve the clinical efficacy, researchers attempted to use acupuncture combined with moxibustion to treat insomnia after stroke, and finally achieved positive results.^[23–25]^ However, no systematic review has been conducted to evaluate the effectiveness and safety of acupuncture combined with moxibustion so far. Therefore, it is necessary to carry out a systematic review for exploring the concluding evidence of the effectiveness and safety of acupuncture combined with moxibustion in treating patients with insomnia after stroke as clinical guidance.

### 1.5 Objectives

To systematically evaluate the effectiveness and safety of acupuncture and moxibustion in treating insomnia after stroke.

## 2 Methods

### 2.1 Study registration

This protocol has been registered at PROSPERO (http://www.crd.york.ac.uk/PROSPERO) and its registration number is CRD42020216720. Any significant amendments of this protocol will be recorded in the PROSPERO before the review is completed. This protocol is drafted according to the Preferred Reporting Items for Systematic Reviews and Meta-analysis Protocols (PRISMA-P) statement and the Cochrane Handbook for Systematic Reviews of Interventions.^[33,34]^

### 2.2 Inclusion criteria for study selection

#### 2.2.1 Types of studies

In order to evaluate the effect of acupuncture combined with moxibustion in treating insomnia after stroke, all randomized controlled trials (RCTs) to explore the effectiveness and safety of acupuncture combined with moxibustion in the treatment of insomnia after stroke are eligible for inclusion. There is no restriction on language and publication status. Only data from the first phase of the randomized crossover trials will be included.

#### 2.2.2 Types of participants

Patients with insomnia after stroke, regardless of sex, age, race, educational level, economic status, or source of cases. Studies with validated diagnostic criteria will be included.

#### 2.2.3 Types of interventions and comparisons

All kinds of acupuncture therapy and moxibustion therapy will be included. The kinds of acupuncture therapy mainly include pure acupuncture, electroacupuncture, ear acupuncture, fire acupuncture, acupoint injection, acupoint catgut embedding and the main kinds of moxibustion therapy consist of moxa stick moxibustion, moxa cone moxibustion, and moxibustion with moxibustioner. The following comparisons will be investigated.

1. Acupuncture and moxibustion compared with Chinese herbal medicine;
2. Acupuncture and moxibustion compared with western medicine;
3. Acupuncture and moxibustion compared with placebo treatment;
4. Acupuncture and moxibustion compared with acupuncture alone;
5. Acupuncture and moxibustion compared with moxibustion alone.

If the 2 groups received the same additional active therapy on the basis of control treatment, the study can be also included.

#### 2.2.4 Types of outcome measures

##### 2.2.4.1 Primary outcomes

The Pittsburgh sleep quality index (PSQI) will be assessed as the primary outcome. This scale is mainly used to evaluate the sleep quality, with a total score ranging from 0 to 21 points. A higher score indicates poorer sleep quality.^[35]^

##### 2.2.4.2 Secondary outcomes

The secondary outcomes in the review are the following 3 aspects.

1. Clinical efficacy which was measured by the efficacy standards of Chinese medicine (cured, markedly effective, effective, not effective).
2. Quality of life which was measured by a validated instrument questionnaire (e.g., the 36-Item Short Form Health Survey [SF-36], the World Health Organization QoL [WHOQOL]).
3. Safety evaluation (e.g., adverse events, Treatment Emergent Symptom Scale [TESS]).

### 2.3 Search methods for identification of studies

#### 2.3.1 Electronic searches

Two independent reviewers (SJJ and ZZ) will search the following eight databases from the inception to October 2020, including China National Knowledge Infrastructure Database (CNKI), Chinese Scientific Journal Database (VIP database), China Biomedical Literature Database (CBM), Wanfang Data Chinese Database, PubMed, the Cochrane Central Register of Controlled Trials (CENTRAL), Allied and Alternative Medicine Database (AMED), Excerpt Medica Database (Embase). There are no restrictions on language and publication status. The combined method of MeSH Term and free words will be used for literature retrieval. A search strategy of PubMed is shown in Table 1, which is created on the basis of the Cochrane handbook guidelines. The search strategies of other databases will be established similarly.

**Table 1:** Search strategy used in PubMed.

No	Search items
#1	((randomized controlled trial[PT] OR controlled clinical trial[PT]) OR randomized[Ti/ Ab]) OR placebo[Ti/Ab] OR“clinical trials as topic”[MeSH Terms] OR randomly[Ti/Ab] OR trial[Ti] NOT (animals[MeSH Terms] NOT (humans[MeSH Terms]) AND animals[MeSH Terms])
#2	Acupuncture[Ti/Ab] OR Acupuncture-moxibustion[Ti/Ab] OR Needle[Ti/Ab] OR Acumoxibustion[Ti/Ab] OR Acupunctural[Ti/Ab] OR Needling[Ti/Ab] OR Acupuncturing[Ti/Ab] OR Electroacupuncture[Ti/Ab] OR Electro-acupuncture[Ti/Ab] OR “Acupoint Injection”[Ti/Ab] OR “Auricular Plaster”[Ti/Ab] OR “Ear Seed Pressure”[Ti/Ab] OR Embedding[Ti/Ab] OR Percussopuncture[Ti/Ab] OR “Point Injection”[Ti/Ab] OR “Pricking Blood”[Ti/Ab] OR “Trigger Points”[Ti/Ab] OR Meridians[Ti/Ab] OR Acupuncture-moxibustion [MeSH Terms] OR Acupuncture [MeSH Terms] OR Acupuncture Therapy [MeSH Terms] OR Electroacupuncture [MeSH Terms]
#3	Moxibustion[Ti/Ab] OR Moxa[Ti/Ab] OR Mox∗[Ti/Ab] OR Mugwort[Ti/Ab] OR Moxibustion [MeSH Terms]
#4	#2 AND #3
#5	Stroke[Ti/Ab] OR Apoplexy[Ti/Ab] OR CVA[Ti/Ab] OR “Cerebrovascular Attack” [Ti/Ab] OR “Cerebrovascular Accident” [Ti/Ab] OR “Cerebral Infarction” [Ti/Ab] OR “Cerebral Hemorrhage” [Ti/Ab] OR Stroke [MeSH Terms]
#6	Insomnia[Ti/Ab] OR “Sleep Disorders”[Ti/Ab] OR Sleeplessness OR Insomnia [MeSH Terms] OR “Sleep Disorders” [MeSH Terms] OR “Sleep Initiation and Maintenance Disorders” [MeSH Terms]
#7	#5 AND #6
#8	“Post-stroke Insomnia” [Ti/Ab] OR “Insomnia after Stroke” [Ti/Ab]
#9	#7 OR #8
#10	#1 AND #4 AND #9

#### 2.3.2 Searching other resources

We will also search for ongoing trials from mainstream registries: the World Health Organization International Clinical Trial Registration Platform search portal (www.who.int/trialsearch/); Chinese Clinical Trial Register (www.chictr.org); Clinical trials.gov (http://ClinicalTrials.gov). In addition, the reference lists of studies meeting the inclusion criteria will also be searched for achieving the comprehensive retrieval to the maximum.

### 2.4 Data collection and analysis

#### 2.4.1 Selection of studies

**Figure 1:**
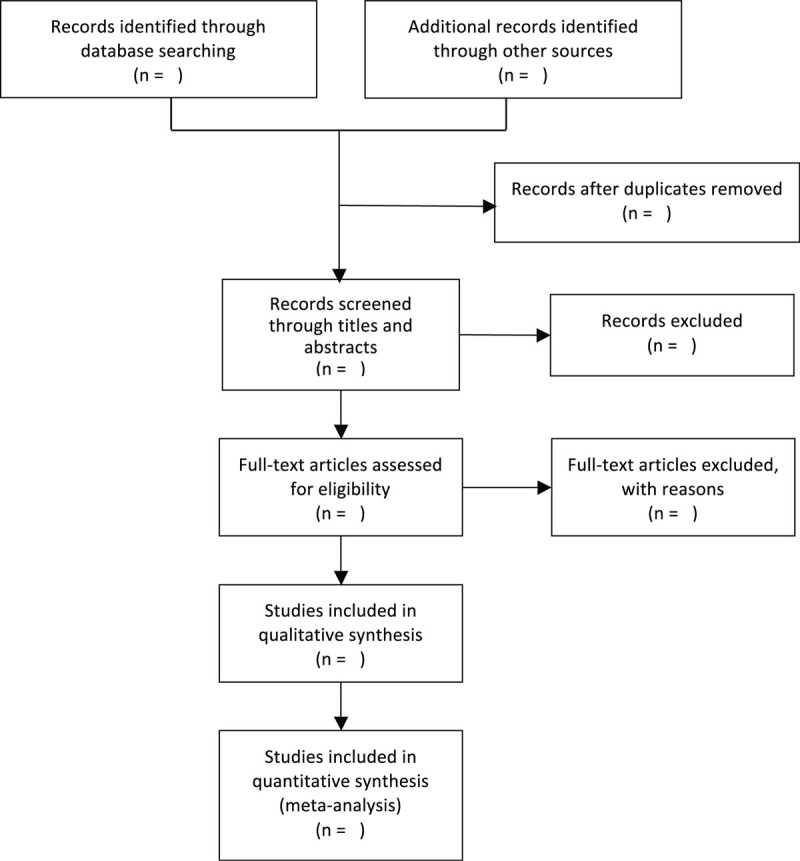
Flow diagram of the study selection process.

We will first use NoteExpress software (V.3.2) to remove duplicates, and then screen the retrieved studies separately by 2 reviewers (SJJ and ZZ) according to the inclusion criteria. On the basis of the article type, clinical characteristics of subjects, and interventional measures, the 2 reviewers (SJJ and ZZ) will independently read the title and abstract of all retrieved studies for preliminary screening. During the process of preliminary screening, the inclusion criteria should be lowered to avoid screening out by mistake. The full text of the potentially relevant studies will be downloaded for final screening. Any disagreement between the 2 reviewers (SJJ and ZZ) will be resolved by a discussion. Further disagreements will be arbitrated by the third author (ZMS). If insufficient or ambiguous data exist, the authors of the original studies will be contacted through an email or telephone to request the additional information or clarification. For the repeatedly published articles, the one with the latest published year or the largest amount of trial information will be included. The process of the selection is shown in Fig. 1.

#### 2.4.2 Data extraction and management

We will firstly design an extraction form that meets the purpose of this system review, which will include the following information of the included studies: the basic information, the characteristics of subjects, the details of interventions, outcomes, methodological section, and adverse events. Before formal extraction, a pretest will be conducted. If deficiencies are found, the data extraction form should be improved in time if any defect exists. The 2 reviewers (SJJ and ZZ) will independently extract data and cross-check the extracted information. Missing data will be obtained by contacting the authors of the original studies. Any disagreement will be resolved through discussion between the 2 reviewers (SJJ and ZZ) or consultation with the third author (ZMS).

#### 2.4.3 Assessment of risk of bias in included studies

According to version 2 of the Cochrane risk-of-bias tool, the methodological quality of RCTs will be independently evaluated by 2 reviewers (SJJ and ZZ), which mainly includes 5 domains of bias: bias arising from the randomization process, bias due to deviations from intended interventions, bias due to missing outcome data, bias in measurement of the outcome, and bias in selection of the reported result. The judgements will be assigned into 3 levels: low risk of bias, high risk of bias, or some concerns. Any disagreement between the 2 reviewers (SJJ and ZZ) will be resolved by a discussion. Further disagreements will be arbitrated by the third author (ZMS).

#### 2.4.4 Measures of treatment effect

For dichotomous data, we will adopt relative risk (RR) with 95% confidence intervals (CIs) to measure the treatment effect. Mean difference (MD) or standard mean difference (SMD) with 95% CIs will be chosen to measure the treatment effect of continuous data.

#### 2.4.5 Dealing with missing data

We will try our best to contact the authors of the original studies to request missing data. If the data cannot be obtained, we will use an intention-to-treatment (ITT) analysis, in which all the randomly assigned subjects will be included in the data analysis regardless of whether they are finally dropped out or lost to follow-up. It is assumed that outcome events have occurred in all subjects of drop-out, withdrawal, or loss to follow-up. At the same time, sensitivity analysis will be carried out to evaluate the influence of missing data replacement on the results and the stability of the results under the assumed conditions. In the discussion part of system review, the potential influence of missing data on the results will be explained.

#### 2.4.6 Assessment of heterogeneity and data synthesis

We will assess clinical heterogeneity of the studies by the variability in subjects, interventions, and outcomes firstly. If there is no clinical heterogeneity, statistical heterogeneity between studies will be further assessed using the Chi^2^ test. Meta-analysis will be performed using RevMan 5.4.1 statistical software. If the *I*^2^ value is <50%, heterogeneity will be considered not important, then fixed-effects model will be used for data synthesis. If the *I*^2^ value is between 50% and 75%, it may represent moderate heterogeneity and the random-effects model will be adopted for meta-analysis. If the *I*^2^ value exceeds 75%, it is considered that there is considerable heterogeneity, and we will conduct subgroup analysis to explore the possible causes of heterogeneity, or systematically describe the characteristics and findings of the included study.

#### 2.4.7 Assessment of reporting biases

If sufficient studies are included (at least 10), we will test the reporting biases in the meta-analysis by using an inverted funnel plot.^[36]^ However, funnel plot asymmetry is not completely equivalent to reporting bias. Given the difficulties in detecting and correcting reporting bias, all studies that met the inclusion criteria will be comprehensively retrieved, including unpublished studies, and the trial registries will also be used to minimize reporting bias.

#### 2.4.8 Subgroup analysis

If there are adequate studies and available data, we will conduct subgroup analysis for different kinds of acupuncture and moxibustion and different syndrome types of insomnia to explain the heterogeneity among studies.

#### 2.4.9 Sensitivity analysis

If sufficient studies are available, we will perform sensitivity analyses to confirm the robustness of the primary results. The meta-analysis will be respectively processed by excluding studies with small sample size, low methodological quality or controversy between 2 reviewers. We will compare the results before and after excluding the studies, and discuss if the results vary significantly.

#### 2.4.10 Grading the quality of evidence

The certainty of a body of evidence will be assessed by using the approach developed by the Grades of Recommendation, Assessment, Development and Evaluation Working Group (GRADE Working Group), involving risk of bias, heterogeneity, indirectness, imprecision, publication bias, and other domains. The certainty level will be rated as high, moderate, low, or very low. The strength of evidence recommendation will be judged as strong or weak.^[37]^

## 3 Discussion

Stroke has a high rate of incidence, mortality, disability, and recurrence. Insomnia is one of the common complications in patients with stroke, which can seriously affect the prognosis and quality of life of patients, even cause the recurrence of stroke and increase the social economic burden. At present, drug therapy and cognitive behavioral therapy are mainly used for insomnia patients after stroke. However, long-term use of these drugs will lead to a variety of side effects and patients’ compliance with cognitive behavioral therapy is low. Many patients seek the help of complementary and alternative therapies. Acupuncture and moxibustion, as the representatives, have received extensive attention due to superior efficiency and low cost. A large number of studies have shown that acupuncture combined with moxibustion has a definite effect on insomnia after stroke. However, no systematic review has been conducted on the effectiveness and safety of acupuncture combined with moxibustion in treating the patients with insomnia after stroke. Our systematic review will provide a comprehensive summary of the available evidence for the effectiveness and safety of acupuncture combined with moxibustion in the treatment of insomnia after stroke. We hope that this evidence can help clinicians and health policy-makers make clinical decisions on insomnia after stroke, and bring good news to patients. However, there are some potential limitations in this systematic review. The different kinds of acupuncture and moxibustion methods and different TCM syndrome types of insomnia after stroke in the included studies may bring about great heterogeneity.

## Author contributions

**Conceptualization:** Junjun Sun.

**Data curation:** Zheng Zuo, Ran Song, Xiongying Bao.

**Formal analysis:** Junjun Sun, Zheng Zuo.

**Methodology:** Junjun Sun, Zheng Zuo, Ran Song, Xiongying Bao, Miansheng Zhu.

**Project administration:** Junjun Sun, Miansheng Zhu.

**Supervision:** Miansheng Zhu.

**Validation:** Junjun Sun, Zheng Zuo, Ran Song, Xiongying Bao, Miansheng Zhu.

**Writing – original draft:** Junjun Sun.

**Writing – review & editing:** Zheng Zuo, Ran Song, Xiongying Bao, Miansheng Zhu.
